# Evaluation of the model malaria elimination strategy in Mandla district along with its neighbouring districts: a time series analysis from 2008 to 2020

**DOI:** 10.1186/s12936-023-04477-7

**Published:** 2023-02-06

**Authors:** Mrigendra P. Singh, Harsh Rajvanshi, Praveen K. Bharti, Himanshu Jayswar, Srinath Singh, R. K. Mehra, Manoj Pandey, Ram Shankar Sahu, Brajesh Patel, Ramji Bhalavi, Sekh Nisar, Harpreet Kaur, Aparup Das, Davidson H. Hamer, Altaf A. Lal

**Affiliations:** 1 Malaria Elimination Demonstration Project, Mandla, Madhya Pradesh India; 2 Indian Council of Medical Research – National Institute of Research in Tribal Health (ICMR-NIRTH), Jabalpur, Madhya Pradesh India; 3 Directorate General of Health Services, Government of Madhya Pradesh, Bhopal, Madhya Pradesh India; 4 Department of Health Services, Government of Madhya Pradesh, Mandla, Madhya Pradesh India; 5 Department of Health Services, Government of Madhya Pradesh, Dindori, Madhya Pradesh India; 6 Department of Health Services, Government of Madhya Pradesh, Balaghat, Madhya Pradesh India; 7 Indian Council of Medical Research, Department of Health Research, Ministry of Health and Family Welfare, Government of India, New Delhi, India; 8 Department of Global Health, Boston University School of Public Health, Boston, MA USA; 9 Section of Infectious Diseases, Department of Medicine, Boston University School of Medicine, Boston, MA USA; 10 Foundation for Disease Elimination and Control of India (FDEC India), Mumbai, Maharashtra India; 11 Present Address: Asia Pacific Leaders Malaria Alliance (APLMA), Singapore, Singapore; 12 Present Address: Indian Council of Medical Research – National Institute of Malaria Research (ICMR-NIMR), New Delhi, India; 13 Present Address: Department of Health and Family Welfare, NHM Raigarh, Chhattisgarh, India

**Keywords:** Malaria elimination, Trend of malaria, Malaria Elimination Demonstration Project, Time series, Forecast, Causal effect

## Abstract

**Background:**

Compared to 2017, India achieved a significant reduction in malaria cases in 2020. Madhya Pradesh (MP) is a tribal dominated state of India with history of high malaria burden in some districts. District Mandla of MP state showed a considerable decline in malaria cases between 2000 and 2013, except in 2007. Subsequently, a resurgence of malaria cases was observed during 2014 and 2015. The Malaria Elimination Demonstration Project (MEDP) was launched in 2017 in Mandla with the goal to achieve zero indigenous malaria cases. This project used: (1) active surveillance and case management using T4 (Track fever, Test fever, Treat patient, and Track patient); (2) vector control using indoor residual sprays and long-lasting insecticidal nets; (3) information education communication and behaviour change communication; and (4) regular monitoring and evaluation with an emphasis on operational and management accountability. This study has investigated malaria prevalence trends from 2008 to 2020, and has predicted trends for the next 5 years for Mandla and its bordering districts.

**Methods:**

The malaria prevalence data of the district Mandla for the period of January 2008 to August 2017 was obtained from District Malaria Office (DMO) Mandla and data for the period of September 2017 to December 2020 was taken from MEDP data repository. Further, the malaria prevalence data for the period of January 2008 to December 2020 was collected from DMOs of the neighbouring districts of Mandla. A univariate time series and forecast analysis was performed using seasonal autoregressive integrated moving average model.

**Findings:**

Malaria prevalence in Mandla showed a sharp decline [− 87% (95% CI − 90%, − 84%)] from 2017 to 2020. The malaria forecast for Mandla predicts zero cases in the next 5 years (2021–2025), provided current interventions are sustained. By contrast, the model has forecasted a risk of resurgence of malaria in other districts in MP (Balaghat, Dindori, Jabalpur, Seoni, and Kawardha) that were not the part of MEDP.

**Conclusion:**

The interventions deployed as part of MEDP have resulted in a sustainable zero indigenous malaria cases in Mandla. Use of similar strategies in neighbouring and other malaria-endemic districts in India could achieve similar results. However, without adding extra cost to the existing intervention, sincere efforts are needed to sustain these interventions and their impact using accountability framework, data transparency, and programme ownership from state to district level.

## Background

India has reported a 46% reduction in malaria cases during 2019–2020 relative to pre-COVID-19 pandemic years. This reduction was mainly attributed to the preparedness, response, and integration of malaria services into COVID-19 services [1, 2]. However, among the 11 High Burden to High Impact (HBHI) countries, India has reported a reduction in malaria cases during–2020. This achievement was attributed to uninterrupted community-based fever surveillance [3]. India is a federal union of a total of 36 states and union territories. The central state of Madhya Pradesh (MP) consists of 14.65% of the country’s total tribal population [4, 5]. Malaria control in this state is challenging due to complex epidemiology such as presence of *Plasmodium falciparum* and *Plasmodium vivax* species with seasonal variations, perennial and seasonal transmission, *Anopheles culicifacies* and *Anopheles fluviatilis* are main vectors, historical resistance to anti-malarial drugs and insecticides, topography, poor socio-economic indicators, and inadequate understanding of socio-behavioural factors [6–8]. Mandla is a tribal dominated hilly and forested district in the state of MP, which was selected for the elimination demonstration project because of high malaria endemicity [9].

The historical trend of malaria prevalence in Mandla revealed a considerable decline (91.88%) from 12,739 malaria cases in 2000 to 1035 malaria cases in 2013, except in 2007 (5547 malaria cases). However, a resurgence of malaria cases during 2014 (3020 malaria cases) and 2015 (4018 malaria cases) was observed. In 2016, the Indian Council of Medical Research, Government of Madhya Pradesh, and the Foundation of Disease Elimination and Control of India, a Community Social Responsibility (CSR) subsidiary of Sun Pharmaceutical Industries Ltd. conceptualized the MEDP in Mandla district through a public private partnership model [9]. The goal of MEDP was to demonstrate achievement of zero indigenous malaria cases and develop a replicable model for other parts of the country and high-burden geographies around the world.

The MEDP used: (1) robust active surveillance and case management using T4 (Track fever, Test fever, Treat patient, and Track patient) [10]; (2) vector control using indoor residual spraying (IRS) and long-lasting insecticidal nets (LLINs) [11, 12]; (3) intensive Information education communication and behaviour change communication [13, 14]; (4) regular monitoring and evaluation [15]; (5) real time data management and data driven quick response [16]; and (6) uninterrupted supply chain management [9, 10]. The field operations of the project began in June 2017 and ended in March 2021. As of May 2020, the project achieved a reduction of 91% of indigenous malaria cases in Mandla district [8] and zero indigenous cases were documented in nine out of 46 months of total project operations [9]. During the COVID-19 pandemic, MEDP was granted ‘emergency services’ status during the first lockdown in March 2020, which continued through all lockdowns and restrictions imposed due to the COVID-19 pandemic. This status ensured that the fever surveillance activities and malaria treatment services remained uninterrupted in the entire district. Zero indigenous malaria cases were also documented up-to four consecutive months from December 2020 to March 2021, which was the post-monsoon malaria transmission season in the district [10, 17].

This study aims to present the trend of malaria prevalence from 2008 to 2020 and predict trends for the next 5 years of district Mandla and its bordering districts using univariate time series models. Additionally, the impact of MEDP interventions was analysed and compared with malaria endemic bordering districts. This forecasting is expected to assist with modification of existing malaria elimination strategies in the study area.

## Methods

**Fig. 1:**
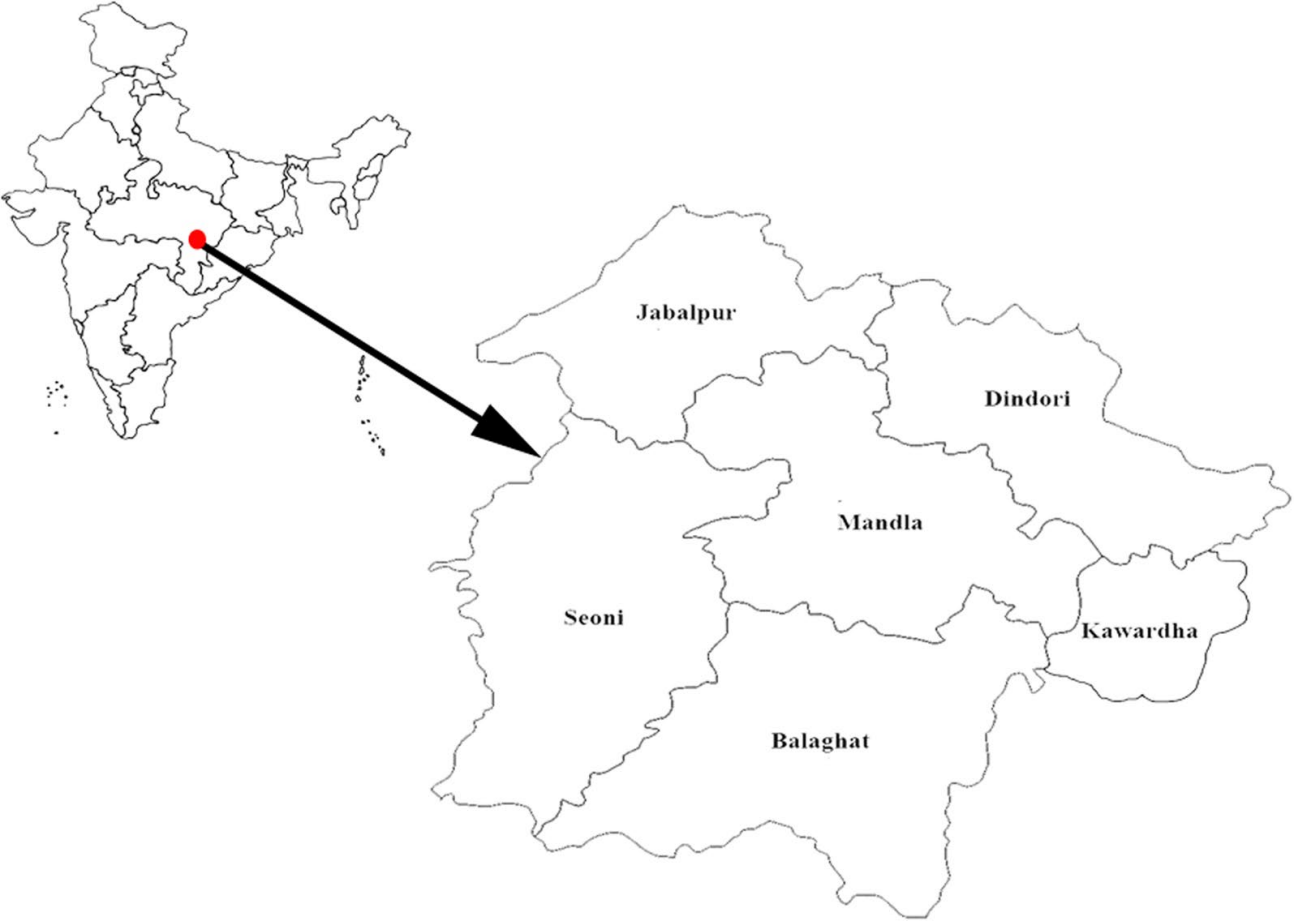
Map of India (in inset) showing Mandla, Balaghat, Dindori, Jabalpur, Seoni districts of Madhya Pradesh state and Kawardha district of Chhattisgarh state (map not to scale)

This is a quantitative analytical study using monthly malaria prevalence (slide positivity rate) data for the period of 2008 to 2020. The reported monthly malaria data consisting total number of fever cases screened, malaria positive cases, *Plasmodium falciparum*, *Plasmodium vivax*, mixed infection of *P. falciparum* and *P. vivax*, slide positivity rate (number of malaria positive per hundred fever cases screened), and proportion of *P. falciparum* and *P. vivax* was collected from the District Malaria Office of the State Vector Borne Control Programme in Mandla for the period from January 2008 to August 2017 (pre MEDP period). The MEDP reported malaria prevalence data for the period from September 2017 to December 2020 was added in the state reported malaria data of Mandla district, completing the Mandla dataset from January 2008 to December 2020. There was no duplication in malaria cases data collected from DMO and MEDP in Mandla district. State reported malaria prevalence data for the same period (January 2008 to December 2020) was collected from bordering districts of Balaghat, Dindori, Jabalpur, Seoni and Kawardha (Fig. 1).

### Study area and population

Mandla district is geographically situated between 22.63 N latitude and 80.54 E longitude. About 45% of the total geographical area is forest covered, average annual rainfall is 1200 mm, and rice is the main crop in the district. As per census 2011, the total population of the district Mandla was 1.055 million, of which 12% population were residing in the urban areas and total literacy was 57%. About 58% population belonged to the ethnic tribal groups mainly “*Gond*” and “*Baiga*”.

Balaghat district is geographically situated between 21.86 N latitude and 80.37 E longitude with 53% area forest cover, and average annual rainfall is 1300 mm. Paddy field (rice) is the main agriculture crop in the district. As per census 2011, the total population was 1.702 million, of which 14% population were residing in the urban areas and total literacy was 67%. About 23% population belonged to the ethnic tribal groups mainly “*Baiga*”.

Dindori district is geographically situated between 22.85 N latitude and 81.07 E longitude with 40% area forest cover, and average annual rainfall is 1450 mm. Paddy field (rice) is the main agriculture crop in the district. As per census 2011, the total population was 0.705 million, of which 5% population were residing in the urban areas and total literacy was 54%. About 65% population belonged to the ethnic tribal groups mainly “*Baiga*”.

Jabalpur district is geographically situated between 23.35 N latitude and 80.01 E longitude with 21% area forest coverage, and average annual rainfall of 1400 mm. Wheat, maize and pulses are the main agriculture crops in the district. As per census 2011, the total population was 2.463 million, of which 58% population were residing in the urban areas and total literacy was 71%. About 15% population belonged to the ethnic tribal groups mainly “*Gond*”.

Seoni district is geographically situated between 22.47 N latitude and 79.56 E longitude with 35% area forest coverage, and average annual rainfall of 1350 mm. Wheat and maize are the main agriculture crops in the district. As per census 2011, the total population was 1.379 million, of which 12% population were residing in the urban areas and total literacy was 63%. About 38% population belonged to the ethnic tribal groups, mainly “*Gond*”.

Kawardha district is geographically situated between 22.10 N latitude and 81.25 E longitude with 37% area forest coverage, and average annual rainfall of 1200 mm. Rice is the main agriculture crop in the district. As per the 2011 census, the total population was 0.823 million, of which 11% population resided in urban areas, and total literacy was 50%. About 20% of the population belonged to the ethnic tribal groups, mainly “*Gond*” and “*Baiga*”.

*Plasmodium falciparum* and *P. vivax* are the two dominant species with seasonal variation and *Anopheles culicifacies* is the primary malaria vector in the study districts. Unlicensed medical practitioners were the most preferred health providers in the community [18].

### Major interventions in the study area

During the period of 2008 to 2020 the major interventions introduced in the national malaria programme were (1) Deployment of Accredited Social Health Activists (ASHA) in year 2009 for providing diagnosis and treatment of clinically suspected malaria cases at door step; (2) Distribution of long lasting insecticide nets (LLIN) in high endemic areas from 2009; (3) Monovalent rapid diagnostic test kits (RDT) introduced to diagnose *P. falciparum* and all confirmed *P. falciparum* positive cases treated with artemisinin-based combination therapy (ACT) from year 2010; (4) Introduction of bivalent (Pf/Pv) antigen based RDT for on-spot diagnosis and treatment of clinically suspected malaria cases from the year 2013; (5) Scaling up distribution of LLIN during 2015–2017; (6) National Framework for Malaria Elimination 2016–2030 introduced in 2016.

### Statistical methods

A seasonal autoregressive integrated moving average (seasonal ARIMA) model for time series and forecasting of monthly malaria prevalence data for the period of 2008 to 2020 of Mandla district and its bordering districts Balaghat and Dindori was analysed. Further, yearly malaria prevalence data for the period of 2008 to 2020 of other bordering districts namely Jabalpur, Seoni of Madhya Pradesh and Kawardha of Chhattisgarh state was also analysed using non-seasonal ARIMA model. Monthly seasonal and secular trends in malaria prevalence data were explored using smoothing regression lines for Mandla, Balaghat and Dindori districts. The raw time series data was decomposed to determine the seasonality, trend and remainder (random) distribution over the period.

The notation (p,d,q) x (P,D,Q)S in seasonal ARIMA models for prediction of malaria prevalence described the composition of temporal patterns considered for forecasting; where p = non-seasonal autoregressive (AR) order, d = non-seasonal differencing, q = non-seasonal moving average (MA) order, P = seasonal AR order, D = seasonal differencing, Q = seasonal MA order, and S = time span of repeating seasonal pattern which is ‘12’ in this study. Box-Jenkins approach in selection of best fitted ARIMA model was adapted.

The monthly malaria prevalence data was plotted to examine the stationarity, correlogram and partial correlogram. The auto correlation function (ACF) and partial auto correlation function (PACF) of the residuals were graphically plotted at each time point as the lag. Outliers in the time series response variable were identified and cleaned. Log transformation and one differencing method were applied to stationary time series data. Akaike Information Criterion (AIC) values were compared in varying orders of the model and autocorrelation residuals were tested using the Ljung-Box test [19].

Further, segmented regression in interrupted time series data and causal impact analysis was also performed to test the impact of interventions tools, such as RDTs, ACT, LLINs, and the MEDP at different points in time.

## Results

Mandla, Balaghat, Dindori and Kawardha were among the highly malarious districts in the state. During 2008–2020, the malaria cases ranged between 56–4018, 574–7601, 371–4845, and 461–21522 respectively in Mandla, Balaghat, Dindori and Kawardha districts. While, Jabalpur and Seoni were low malaria endemic districts with the malaria cases ranged between 94–1858 and 102–1575 respectively during 2008–2020 (Table 1).

**Table 1:** Malaria cases in Mandla and bordering districts during 2008—2020

Year	Districts																	
	Mandla			Balaghat			Dindori			Jabalpur			Seoni			Kawardha		
		Positive			Positive			Positive			Positive			Positive			Positive	
	No. tested	N	%	No. tested	N	%	No. tested	N	%	No. tested	N	%	No. tested	N	%	No. tested	N	%
2008	200,111	3200	1.60	230,813	2966	1.29	115,487	1242	1.08	262,338	1200	0.46	166,812	1575	0.94	51,908	863	1.66
2009	196,129	2519	1.28	235,171	2654	1.13	156,611	3546	2.26	270,567	1251	0.46	182,588	1382	0.76	83,542	1293	1.55
2010	180,340	2656	1.47	247,027	1768	0.72	159,268	4201	2.64	275,697	1858	0.67	169,328	1056	0.62	82,114	2234	2.72
2011	215,091	2112	0.98	246,702	1939	0.79	142,512	1463	1.03	313,755	1127	0.36	190,475	1104	0.58	64,326	2098	3.26
2012	225,774	1101	0.49	287,262	2300	0.80	144,749	829	0.57	298,245	508	0.17	186,423	1019	0.55	286,019	21,522	7.52
2013	248,470	1035	0.42	348,767	2594	0.74	131,788	1253	0.95	303,415	478	0.16	191,815	808	0.42	78,971	2906	3.68
2014	254,320	3020	1.19	329,582	7601	2.31	159,055	3243	2.04	324,817	374	0.12	179,316	1199	0.67	91,145	7175	7.87
2015	265,726	4018	1.51	282,478	4829	1.71	183,615	4845	2.64	336,427	372	0.11	175,169	601	0.34	110,167	9194	8.35
2016	225,001	1431	0.64	282,521	3111	1.10	192,035	3454	1.80	326,675	316	0.10	155,299	384	0.25	152,896	4079	2.67
2017	180,786	435	0.24	293,969	1550	0.53	147,373	1331	0.90	326,014	350	0.11	198,913	1569	0.79	97,839	5014	5.12
2018	287,461	330	0.11	267,108	1324	0.50	114,212	1145	1.00	322,344	230	0.07	163,665	483	0.30	159,334	949	0.60
2019	295,190	196	0.07	271,746	1165	0.43	124,761	780	0.63	358,391	94	0.03	175,925	102	0.06	221,204	461	0.21
2020	180,381	56	0.03	274,054	574	0.21	105,065	373	0.36	NA	NA	NA	NA	NA	NA	NA	NA	NA

*NA* Not available

### Seasonal and yearly variability

A seasonal trend with highest malaria prevalence in the month of November and lowest in the month of March was observed in monthly time series malaria prevalence data from Mandla and Balaghat districts during the years 2008–2020. In Dindori, most of the malaria cases were reported during July, August and November and least in the month of April. There were two peaks; one in the months of July–August and another in November in Balaghat and Dindori districts, while Mandla district had only one malaria peak in the month of November.

**Fig. 2:**
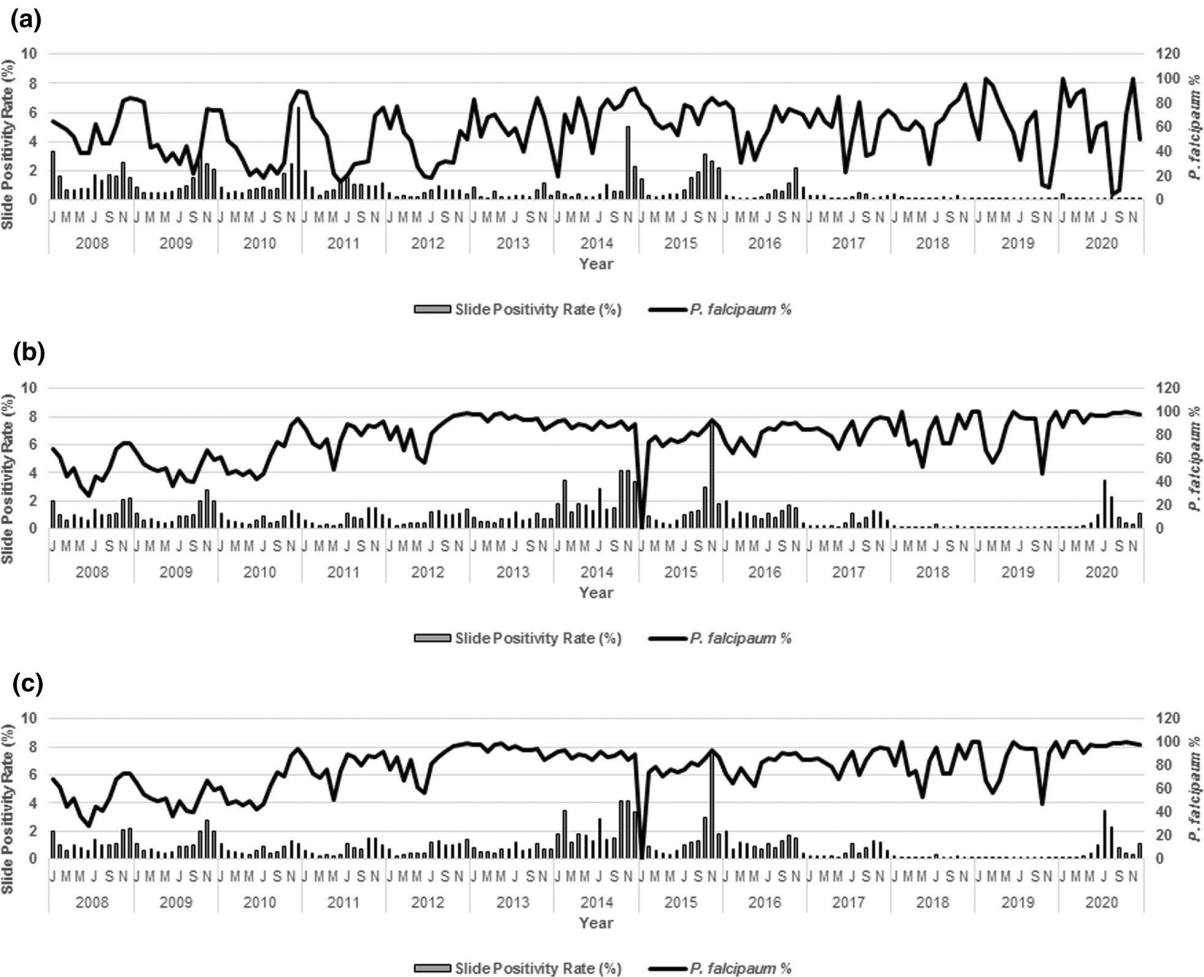
Seasonal trend of malaria during 2008 to 2020 in Mandla, Balaghat and Dindori districts

*Plasmodium falciparum* was the dominant species, which ranged between 73 to 87% of the total malaria cases in Balaghat district. While in Dindori district the *P. falciparum* cases reported during most of the months in a calendar year. However, the proportion of *P. falciparum* cases in Mandla district was highest during October–April and was replaced with *P. vivax* in May to September (Fig. 2).

**Fig. 3:**
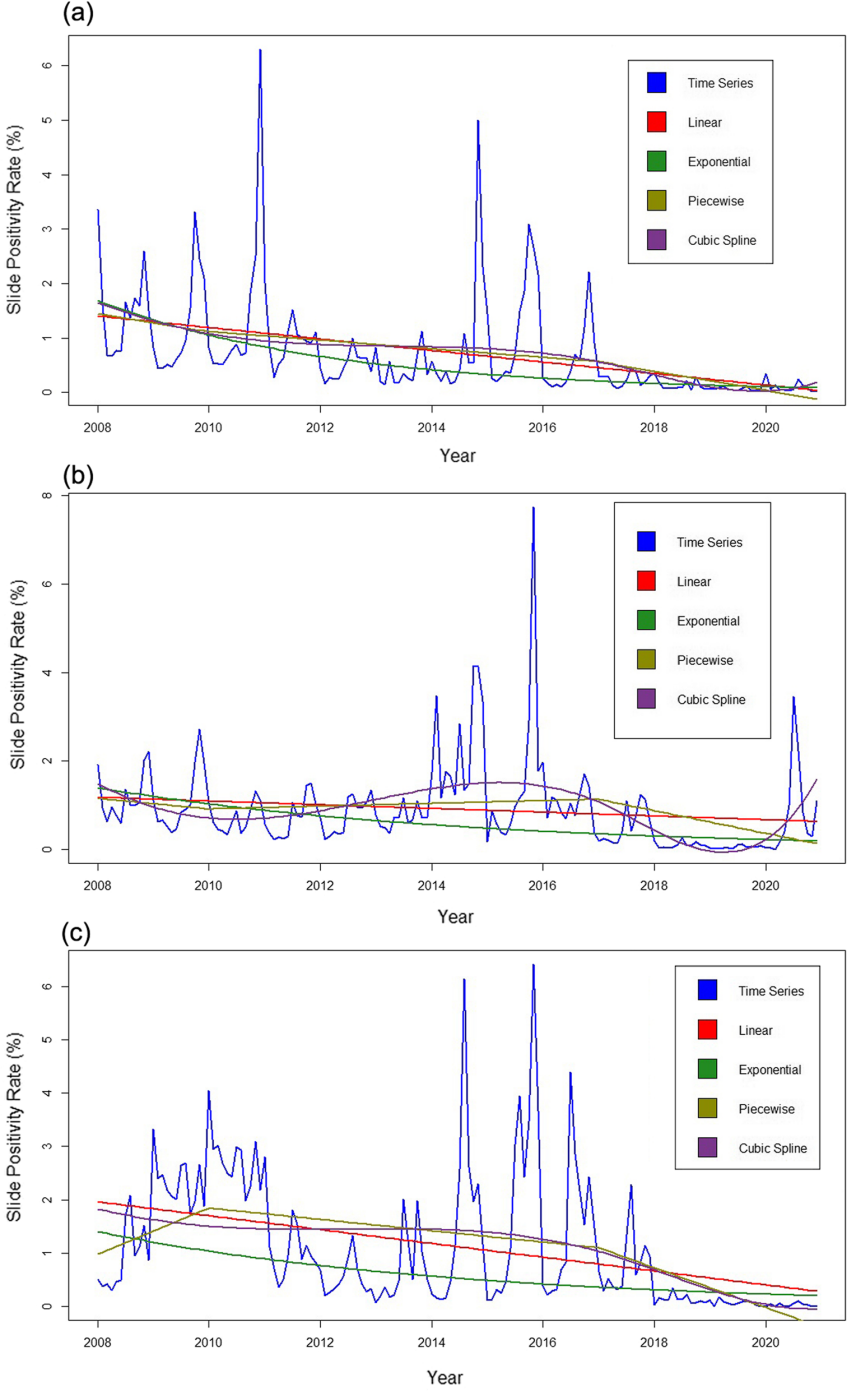
Time series plot of malaria prevalence during 2008 to 2020 for **a** Mandla; **b** Balaghat; **c** Dindori districts

Figure 3 shows time series data with linear, exponential, piecewise and cubic spline smoothing curve of malaria prevalence data of district Mandla, Dindori and Balaghat. Yearly trend of the time series malaria prevalence in Mandla district showed highest cases in the year 2008 which gradually declined till the year 2013 [− 72.4% (95% CI − 64.5, − 79.3%)]. Subsequently, a significant increase in malaria prevalence was recorded during the years 2014 and 2015 [193.5% (95% CI 190.1, 196.8%)] followed by a steady decline till 2016 and a sharp decline after 2017 (Table 1, Fig. 3A). However, in Balaghat and Dindori districts, the trend was different than Mandla district. In Balaghat, the malaria prevalence declined during 2009 and 2010 and remained mostly stable until 2013. Further, there was more than a threefold increase in prevalence in 2014 followed by a sharp decline until 2019. In 2020, the malaria prevalence increased exponentially in Balaghat district (Table 1, Fig. 3B). In Dindori district, there was more than a two-fold increase in malaria prevalence during 2009 and 2010, followed by a steady decline from 2011 to 2013 and an approximately two-fold increase from 2014 to 2016. The prevalence declined steadily till 2020 (Table 1, Fig. 3C). Linear, exponential, piecewise and cubic spline smoothing curve showed a declining trend in districts Mandla and Dindori, but a cyclic trend in district Balaghat.

### Interrupted time series analysis

Segmented regression of interrupted time series analysis for the impact of RDTs for the on-spot malaria diagnosis and ACT for the treatment of *P. falciparum* malaria, which was introduced by the state programme in 2010 in Mandla district, showed a significant decrease in malaria prevalence [− 41% (95% CI − 72%, − 11%)]. However, in Balaghat and Dindori districts, the decrease in malaria prevalence was not significant [− 11% (95% CI − 39%, 14%)] and [− 15% (95% CI − 44%, 13%)], respectively. Further, a significant decrease in malaria prevalence was found after 2015 when LLINs were introduced by the state programme in Mandla, Balaghat and Dindori districts [− 61% (95% CI − 97%, − 25%)]; [− 38% (95% CI − 64%, − 10%)] and [− 45% (95% CI − 77%, − 18%)], respectively.

**Fig. 4:**
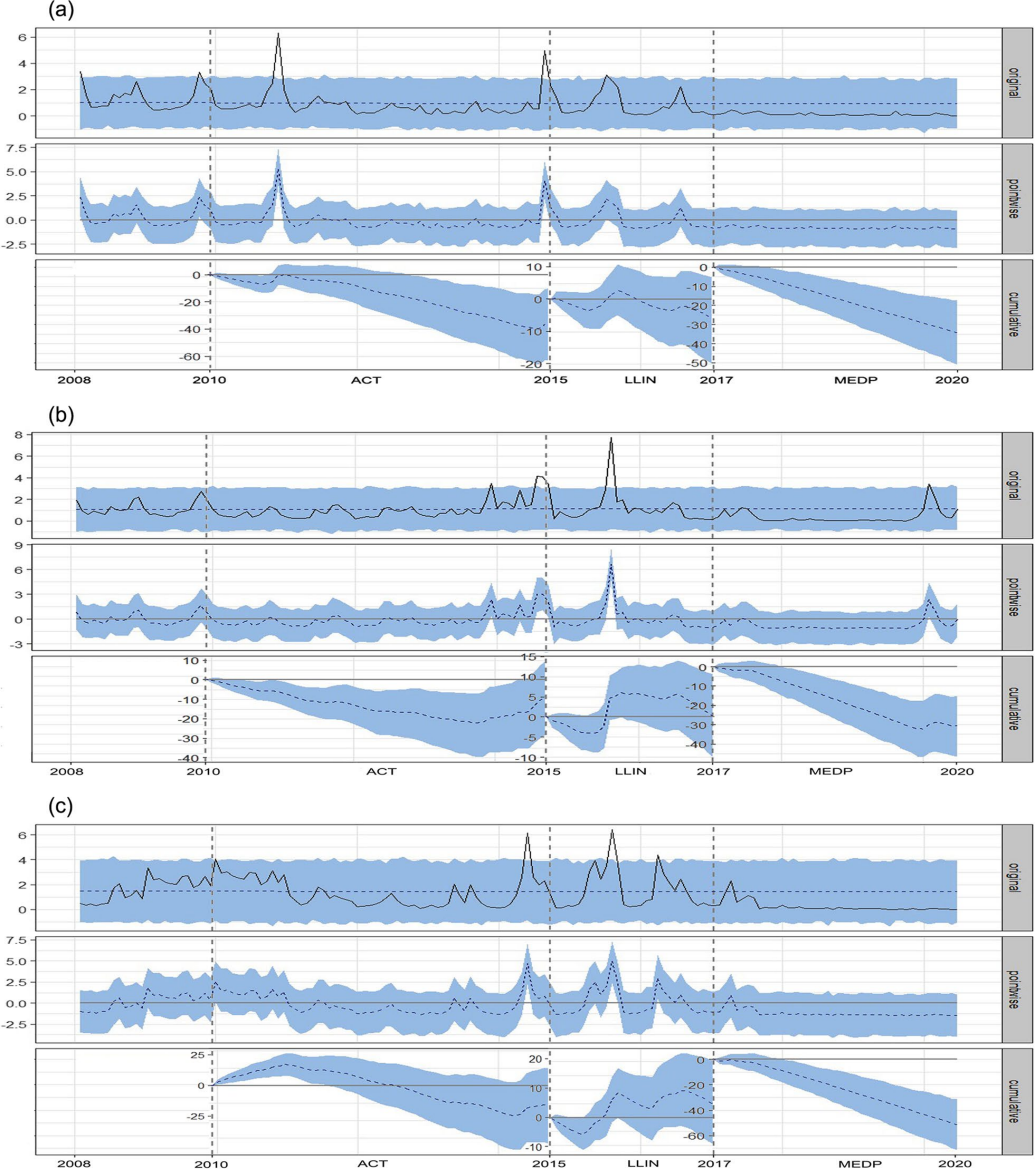
Segmented regression analysis plot showing causal impact of ACT, LLIN and MEDP intervention periods for **A** Mandla; **B** Balaghat; **C** Dindori districts. Legend: Blue shaded area represents 95% confidence interval

Causal effect of MEDP interventions initiated in June 2017 in Mandla district revealed a decrease in malaria prevalence between 2017 and 2020 of -87% (95% CI − 90%, − 84%); P = 0.001. During the same period in Balaghat and Dindori districts, which were outside the MEDP study area, there was also a decrease in malaria prevalence of − 63% (95% CI − 65%, − 60%) and − 72% (95% CI − 74%, − 69%), respectively. The MEDP was associated with a significantly higher rate of decrease in malaria prevalence when compared with non-MEDP districts (P < 0.0001) (Fig. 4).

### Time series and forecast analysis

**Fig. 5:**
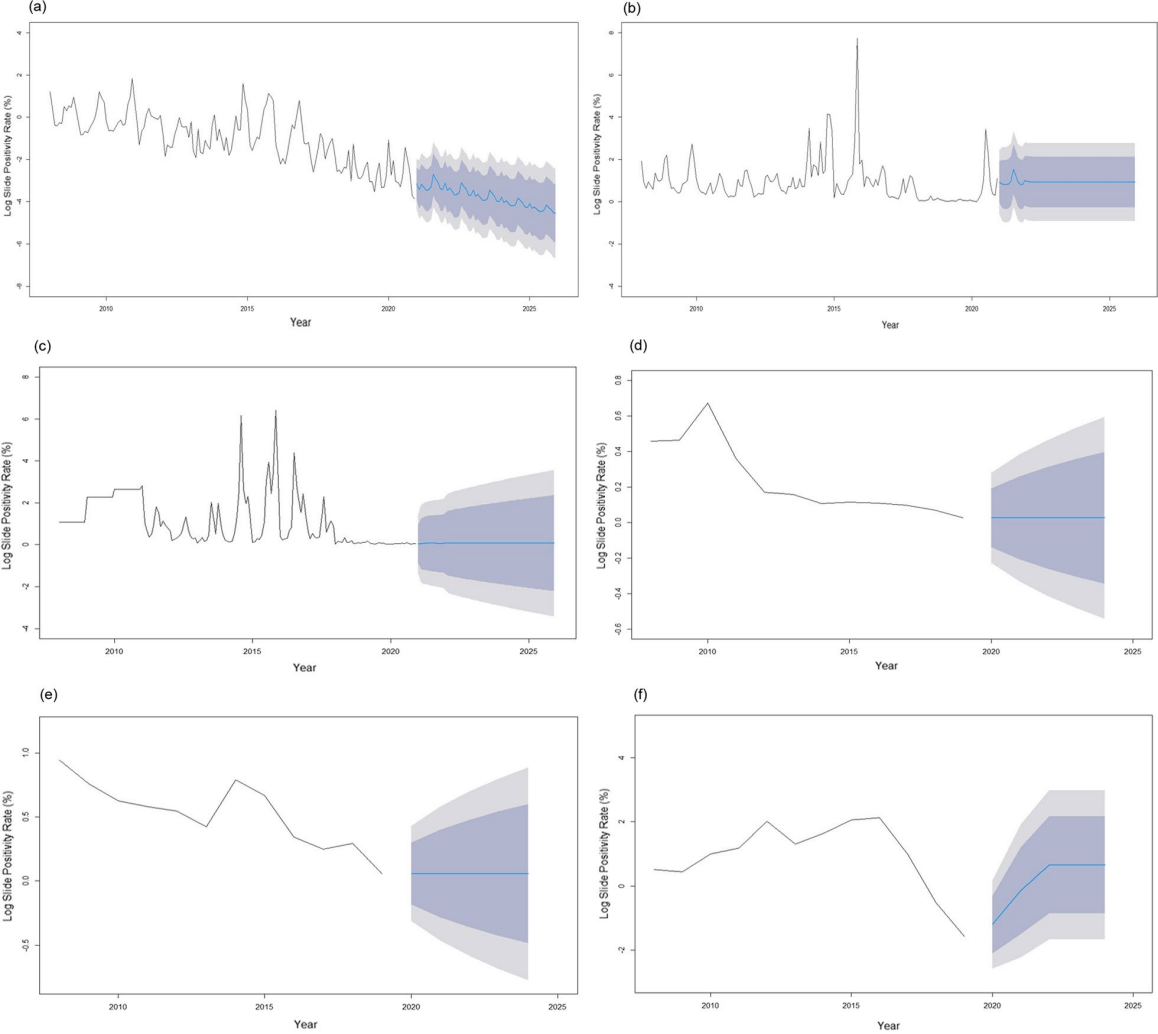
Forecast analysis of malaria prevalence for the period of 2021 to 2025 using ARIMA model for **A** Mandla; **B** Balaghat; **C** Dindori; **D** Jabalpur; **E** Seoni; and **F** Kawardha districts. Legend: Dark gray shaded area represents 80% confidence interval (CI) and light gray shaded area represents 95% CI

Predicted malaria prevalence for the next 5 years (2021–2025) in Mandla district and its bordering districts namely Balaghat, Dindori, Jabalpur, Seoni and Kawardha is shown in Fig. 5. The trend analysis revealed that Mandla district had nearly achieved the malaria elimination goal in 2020 with the 17 indigenous reported malaria cases. The forecast analysis for the next 5 years predicted that Mandla will maintain zero indigenous malaria cases assuming controlled effects of other factors such as robust surveillance with T4 strategy, uninterrupted supplies of diagnostics and antimalarial drugs, optimal coverage of LLIN and IRS, periodic capacity building of healthcare providers, regular IEC and BCC for the community and accountability frameworks, and other external ecological factors. Predictions for Dindori, Jabalpur, and Seoni districts also suggested static zero indigenous malaria cases during 2021–2025; however, the 95% CI was much wider which may not validate the prediction. Balaghat and Kawardha districts are predicted to have unstable malaria transmission, which is likely to increase in subsequent years.

## Discussion

Amidst the COVID-19 pandemic, the worst case scenario for malaria elimination efforts was averted, but urgent action is needed to recover the stalled progress and achieve global malaria targets [20]. Moderate disruptions in the delivery of global malaria services resulted in an increase of 13.4 million cases and 63,000 deaths between 2019 and 2021 worldwide [21]. Nevertheless, there were some malaria elimination success stories during this time. During the pandemic, China and El Salvador were certified as malaria-free in 2021. The Islamic Republic of Iran and Belize reported zero indigenous cases for three and two consecutive years in 2020, respectively [20].

India has registered a sharp decline of malaria prevalence since 2005 with some fluctuations [20]. However, the epidemiological data reported by the national programme always has discrepancy with the estimated cases by the World Health Organization (WHO) [22]. The National Center for Vector Borne Diseases Control (NCVBDC), formerly known as the National Vector Borne Disease Control Programme (NVBDCP) introduced monovalent RDT for diagnosis of *P. falciparum* in 2010 followed by bivalent (*P. falciparum* and *P. vivax*) RDT in 2013 [23]. At the same time, NCVBDC also introduced ACT for treatment of *P. falciparum* cases in 2009–2010 [23]. As a result, diagnosis and treatment of malaria cases, particularly in hard to reach tribal dominant and forested areas where *P. falciparum* was responsible for most malaria cases, have improved. The principal vector control tool, LLINs were introduced in the national programme during 2009–10 [24]; however, significant coverage in hard-to-reach areas was achieved after 2015. Efforts are being made to cover the entire population residing in areas with annual parasite incidence (API) more than one. This will phase out IRS as a key vector control tool and limit its scope to focal spraying.

In India, the highest number of malaria cases are reported during the months of July to October [25]. In Mandla district, the study observed peak of malaria cases in the month of November and the peaks of malaria cases were observed in July, August, and November in the adjoining districts of Balaghat and Dindori. Singh and Sharma have previously reported the prevalence of *P. falciparum* peaked from August to December and *P. vivax* peaked from March to September in Madhya Pradesh [26].

In the present study, the trend of *P. falciparum* and *P. vivax* in Mandla was not similar to the trend observed in rest of the state of Madhya Pradesh. The dominance of *P. falciparum* infection in the months of October to April could be due to the fact the rainfall in October creates favourable conditions for good vegetation and retention of optimal humidity that is required for breeding of mosquitoes in the subsequent months. The switch in trend to *P. vivax* in the months of May to September could be due to resurgence of dormant parasites in the human hosts because in these months, the heavy rainfall washes out the parasite reservoirs from the environment [25].

Mandla district experienced a sharp decline in malaria prevalence from 2017, which was the same year MEDP interventions were implemented in the district. The MEDP interventions achieved a 91% reduction in indigenous malaria cases between June 2017 to May 2020 [10]. However, the overall reduction in all reported malaria cases including migratory population was 87% during June 2017 to December 2020. A declining trend was noted in Balaghat district, which was interrupted with an exponential increase in the year 2020. This increase in malaria cases was an expected outcome amidst the disruption of services due to the COVID-19 pandemic [27]. It should be noted that Mandla was able to preserve its gains during the pandemic. The reason behind this success was the uninterrupted delivery of malaria services and integration of COVID-19 services within the existing delivery model to cater to the entire population of the district [2]. The project has also developed a sustainable and replicable model for malaria elimination from its learnings and experiences in Mandla district. The model has been designed to seamlessly integrate into the existing health systems with no additional costs [28].

A steady reduction in malaria prevalence was noted in Dindori district from 2016 to 2020. This district is inhabited primarily by a particularly vulnerable tribal group (PVTG) known as “*Baiga”* and has achieved 89% reduction in malaria cases during 2009 – 2014 with IRS, LLIN, robust surveillance, and intensive IEC [29]. However, it was interesting to note that despite having a fever case burden similar to Mandla and Balaghat districts, Dindori reported zero malaria cases in the past several months (unpublished data). This finding raises the question of the aetiology of these fever cases in the absence of malaria in the entire district. In Kawardha district, the prevalence gradually increased between 2008–2017, furthermore it was steady decreasing, and the trend was cyclic rather than linear. The non-seasonal ARIMA forecast model predicted an increasing trend in malaria prevalence during next 5 years.

The analysis of the introduction of interventions, such as ACT, bivalent RDTs, LLINs at varying points in time had the expected effect of being associated with decreasing malaria prevalence, similar to findings from other studies conducted in similar areas [10, 29–32]. In 2021, a needs assessment study for Accredited Social Health Activists (ASHAs) of Mandla, Balaghat, and Dindori was conducted (Singh et al. pers. commun.).

In the present study, it was noted that significant malaria reduction was not noted following introduction of ACT and RDTs in Balaghat district, which could be due to poor knowledge about interpretation of correct RDT results and correct identification of ACT age-group-wise combi-packs by the ASHAs of Balaghat. It is interesting to note that the ASHAs of Dindori performed poorer than the ASHAs of Balaghat in this assessment, which once again raises the question of data integrity on near-zero transmission of malaria in Dindori district (Singh et al. pers. commun.).

The promising forecast of zero indigenous transmission of Mandla assuming sustained interventions and unstable malaria transmission in Balaghat and Kawardha has been supported by better and worst cases scenarios, respectively, of the country-level forecasting done by the Bill and Melinda Gates Foundation (BMGF) in their 2021 Goalkeepers Report [33]. The BMGF forecasting used possible disruptions in malaria services due to the COVID-19 pandemic and yielded historical average, better, worse, and 2030 target scenarios [33]. As of 2021, Balaghat is the only district in Madhya Pradesh under category 3 of the National Strategic Plan [34, 35] and Kawardha is a low endemic district with API between zero to one, sharing borders with Dindori [36]. In the pre-COVID times, interventions were tested in these malaria endemic districts with similar topography, epidemiology, and demography. The lack of continuity of these interventions in the study areas of Betul [37], Dindori [29], and Mandla [38] led to a resurgence of malaria, which educates us on the importance of continued and sustained efforts to eliminate malaria.

The WHO conducted a similar forecasting analysis in sub-Saharan Africa which revealed that if the insecticide-treated net campaigns were cancelled and access to treatments was severely disrupted, malaria cases would go up by 23% and deaths by 102% [39]. Another study published by Hogan et al. [40] showed that if there is unmanaged suppression of malaria preventive activities, treatment of clinical cases may be reduced by 50% compared to pre-pandemic levels. In cases of moderate or controlled suppression, this reduction may be halted to 25%. Low-and-middle-income countries could see an increase in deaths due to HIV, tuberculosis and malaria by up to 10%, 20% and 36% as a result of COVID-19.

## Limitations

Data reported in Mandla district during the MEDP period included cases reported in the surveillance systems (both MEDP and government) along with other formal and informal health care providers in the district, whereas malaria cases reported in the bordering districts consisted of only data reported by government health systems [22]. The data reported from Mandla district was subjected to regular spot-inspection and independent audits as part of the overall package of MEDP interventions from 2017. This study used the best-fitting ARIMA model for prediction of malaria prevalence; however, the predicted trends cannot claim the absolute accurate predictions. Changes in geo-climatic condition, socio-cultural and behavioural factors of the community, vector bionomics are the most potential factors in disease transmission and these factors was assumed to be constant in the model. The seasonal model could not be applied due to non-availability of monthly malaria prevalence data for Jabalpur, Seoni, and Kawardha districts.

## Conclusion

The forecasting model of malaria prevalence in Mandla predicts zero cases in the next 5 years, assuming the constant implementation of current intervention strategies and other confounding factors to be controlled. The model suggests that other bordering districts, namely Balaghat, Dindori, Jabalpur, Seoni and Kawardha predict no resurgence of malaria in the next 5 years. However, this prediction comes with poor confidence due to the wide confidence intervals revealed in the model. Therefore, there is an urgent need to integrate the existing intervention strategies with the lessons learnt from the MEDP (e.g., the T4 strategy) and apply them in malaria endemic and tribal dominant forested districts to achieve India’s malaria elimination goal.

## Data Availability

We have reported all the findings in this manuscript. The hardcopy data is stored at MEDP Office in Jabalpur, Madhya Pradesh, and Indian Council of Medical Research-National Institute of Research in Tribal Health (ICMR-NIRTH), Jabalpur, Madhya Pradesh. Softcopy data is available on the project server of MEDP hosted by Microsoft Azure. If anyone wants to review or use the data, they should contact: Dr. Altaf A. Lal, Project Director – Malaria Elimination Demonstration Project, Mandla Foundation for Disease Elimination and Control of India, Mumbai, India 482003, E mail: altaf.lal@sunpharma.com, altaf.lal@gmail.com.

## Abbreviations

ACF: Auto Correlation Function
ACT: Artemisinin Combination Therapy
AIC: Akaike Information Criterion
API: Annual Parasite Incidence
APLMA: Asia Pacific Leaders’ Malaria Alliance
ARIMA: Autoregressive Integrated Moving Average
ASHA: Accredited Social Health Activist
BCC: Behaviour Change Communication
BMGF: Bill and Melinda Gates Foundation
CSR: Corporate Social Responsibility
DMO: District Malaria Office
FDEC: Foundation for Disease Elimination and Control
HBHI: High Burden High Impact
ICMR: Indian Council of Medical Research
IEC: Information Education Communication
IRS: Indoor Residual Spray
LLIN: Long Lasting Insecticidal Net
MEDP: Malaria Elimination Demonstration Project
MP: Madhya Pradesh
NCVBDC: National Center for Vector Borne Diseases Control
NHM: National Health Mission
NIMR: National Insitute of Malaria Research
NIRTH: National Institute of Research in Tribal Health
PACF: Partial Auto Correlation Function
PVTG: Particularly Vulnerable Tribal Group
RDT: Rapid Diagnostic Test
T4: Track Test Treat Track
USA: United States of America
WHO: World Health Organization
